# Case Report: Custom made 3D implants for glenoid tumor reconstruction should be designed as reverse total shoulder arthroplasty

**DOI:** 10.3389/fsurg.2024.1433692

**Published:** 2024-10-16

**Authors:** Robin Evrard, Antoine Ledoux, Pierre-Louis Docquier, Florine Geenens, Thomas Schubert

**Affiliations:** ^1^ Neuro Musculo-Skeletal Lab, Institut de Recherche Expérimentale et Clinique, Université Catholique de Louvain, Bruxelles, Belgium; ^2^ Department of Orthopedic and Trauma Surgery, Cliniques Universitaires Saint Luc, Institut du Cancer Roi Albert II (IRA2), Institut de Recherche Expérimentale & Clinique (IREC), Université Catholique de Louvain (UCLouvain), Brussels, Belgium; ^3^ Service de Médecine Physique et Réadaptation, Cliniques Universitaires Saint-Luc, Bruxelles, Belgium

**Keywords:** glenoid, reconstruction, orthopedic oncology, custom made, case series

## Abstract

**Background and objectives:**

Isolated bone tumors of the glenoid are exceedingly rare occurrence and pose a substantial surgical challenge. 3D printing technology has been proved to be a reliable tool to reconstruct complex anatomical part of the skeleton. We initially used this technology to reconstruct the glenoid component of the shoulder in a hemiarthroplasty configuration. We subsequently changed to a reverse shoulder arthroplasty.

**Methods:**

Two patients were reconstructed with a hemiarthroplasty and 2 with a reverse configuration. Patients files were reviewed for radiographic analysis, pain and function scores.

**Results:**

Mean follow-up was 36.44 ± 16.27 months. All patients are alive and disease free. The two patients who benefitted from a hemiarthroplasty demonstrated a rapid deterioration of the proximal humeral articular surface. Given their pain and function scores, they subsequently required revision towards a total shoulder arthroplasty. Following this conversion, one patient presented a shoulder dislocation requiring surgical reintervention. We did not observe any loosening or infection in this short series.

**Conclusions:**

Custom made glenoid reconstruction should be designed as a reverse shoulder arthroplasty given the mechanical constrains on the proximal humerus and the extent of the surgery invariably damaging the suprascapular neurovascular bundle.

## 1 Introduction

Tumors of the scapula are uncommon occurrences, representing 2.1% of primary benign bone tumors and 3.2% of primary malignant bone tumors occurrences within the human skeleton (1). These tumors pose a unique challenge as they can jeopardize the integrity of the scapulohumeral joint, particularly when they affect the glenoid of the scapula. In cases where the glenoid is exclusively affected and the nature of the tumor necessitates resection with healthy margins, determining the optimal treatment and the choice of reconstruction becomes a critical consideration.

Prosthetic shoulder surgery presents distinctive characteristics in comparison to other major joints that undergo arthroplasty, such as the hip or knee. The shoulder, being the most mobile joint in the human body and suffering any weight-bearing function, is unique. Its stability and function rely significantly on the integrity of the surrounding soft tissues, including the labrum, ligaments, tendons and muscles (2, 3). These factors pose significant challenges in the realm of shoulder arthroplasty. Given its exceptional mobility and crucial functional requirements, it is not uncommon for the shoulder to not fully regain its pre-surgery function (4). In conventional shoulder arthroplasties, reverse total prosthesis became the gold standard when the rotator cuff is compromised, while an anatomical prosthesis is preferred when the rotator apparatus is preserved. It's worth noting that anatomical prostheses have a higher dislocation rate compared to reverse prostheses, representing a notable drawback (5–7).

When performing shoulder surgery, the current consensus advocates for a minimally invasive approach, prioritizing the preservation of bone stock, minimizing incision sizes, and recognizing that the functionality of healthy biological tissue surpasses that of any exogenous implant (8, 9). These principles align with the considerations in oncological surgery. However, surgical planning in tumor surgery remains inherently variable in geometry, adapting on a case-by-case basis. Resections in these scenarios are frequently extensive, necessitating larger and sometimes multiple approaches. Achieving healthy margins often entails a notable reduction in bone stock, and reconstructions commonly require the incorporation of cutting-edge technologies to restore the joint to optimal functionality (10, 11).

In rare cases of malignant or locally destructive tumor specifically impacting the scapular glenoid, reconstruction will often necessitate a massive allograft or a custom-made implant. With the advent of 3D printing of custom-made surgical implants, it has been widely described that these new technologies bring significant added value to patient care in different surgical disciplines, particularly orthopaedics (12–15). The shoulder implants, frequently created through 3D printing based on the patient's pre-operative images, allow for the precise reconstruction of the glenoid with the same anatomical surface as before (11, 16, 17). To the best of our knowledge, shoulder hemiarthroplasty commonly only involves a proximal humerus prosthesis and there is no publication regarding a hemiarthroplasty involving a glenoid custom-made implant in oncological case of glenoid tumors. This approach aligns with the consensus of achieving the least invasive surgery possible by reconstructing only the affected structure and leaving the humerus as it is. This raises the question of whether a custom-made glenoid hemiarthroplasty can genuinely be a superior, or equivalent, solution compared to a custom-made reverse total shoulder arthroplasty.

This article presents a short surgical series involving four patients with a glenoid tumor, where two patients underwent custom-made reverse shoulder arthroplasty, and two underwent custom-made glenoid hemiarthroplasty.

## 2 Cases descriptions

This study is a series of surgical cases concerning shoulder reconstruction after glenoid tumor resection. The clinical information's of 4 patients were collected retrospectively. The agreement of the local ethics committee of the university hospital was obtained to carry out this study (2015/26JAN/025 Belgian registration number B403201523492). Written informed consent was obtained from the individuals and minor's legal guardian/next of kin for the publication of any potentially identifiable images or data included in this article. These four consecutive cases therefore constitute a retrospective monocentric case series study.

Patient 1 is a 16 years old male who suffered from a chondroblastoma and benefited from a custom-made glenoid hemiarthroplasty. Patient 2 is a 60 years old female who suffered from a chondrosarcoma and benefited from a custom-made glenoid hemiarthroplasty. Patient 3 is a 37 years old male who suffered from a chondrosarcoma and benefited from a custom-made reverse total shoulder arthroplasty. Patient 4 is a 58 years old male who suffered from a glenoid prostate carcinoma solitary metastasis and benefited from a custom-made reverse total shoulder arthroplasty.

These four patients all experienced oncological pathology affecting their glenoid structure between 2018 and 2022, with no invasion into the scapulohumeral joint and a preserved humerus. Presently, all four patients are alive and disease-free. The mean follow-up duration was 36.44 ± 16.27 months, with a range of 24.07–60.13 months. The tumor resection surgeries for all four patients were conducted by the same surgical team, employing a consistent operating protocol. Patients were positioned in lateral decubitus. The initial step involved a posterior approach to the scapula, aimed at liberating the scapular spine from the posterior fibers of the deltoid. Subsequently, the infraspinatus tendon was cut 1 cm from its humeral insertion to expose the glenoid. This was followed by a second, conventional deltopectoral approach to release the proximal tendon of the triceps, further isolating the glenoid, and securing the vascular-nerve structures.

The utilization of 3D-printed cutting guides applied form the posterior approach ensured the achievement of safe resection margins (R0) in all four patients. These margins were subsequently confirmed by the pathologist. For patients 1 and 2, a custom-made glenoid hemiarthroplasty, based on pre-operative imaging of the contralateral shoulder, was implanted. In the case of patients 3 and 4, a custom-made reverse shoulder arthroplasty was implanted. All implants were bought from Implantcast, Buxtehude, Germany. The design of the bone anchor onto the shoulder was conceived in collaboration with their custom-made engineering department (Supplementary Figure S1a). The glenoid component was extended with a large flange that applied onto the anterior aspect of the lateral column of the shoulder blade. A second flange covering the posterior aspect of the lateral column was fixed from posterior to anterior with screws in the main body of the implant. All bone contact parts of the implants were designed with a porous titanium surface (EPORE®). This clamp construct with an anterior and posterior flange resulted in a very strong primary stabilization of the implant. The secondary bone in growth in the porous titanium is expected to offer very long-term fixation.

Standard immediate follow-up procedures, including antibiotic prophylaxis, physiotherapy, and length of hospital stay, were consistent across all patients. Immediate mobilization was initiated to facilitate the recovery of ranges of motion (ROM), with a restriction on rotational movements for the initial 4 weeks. Notably, no acute postoperative complications, such as infection, fracture, or dislocation, were observed. When a glenoid hemiarthroplasty was conducted, the quality of the humeral head was confirmed through conventional imaging and macroscopic assessment perioperatively.

Following our clinical and radiological observations on patient 1 and 2, we changed our reconstruction technique for a total reverse shoulder prosthesis construct with a custom-made glenoid component. The surgical technique remained the same with the exception of the additional implantation of a standard reverse humeral stem.

Specific details merit attention for certain patients. In the case of Patient 1, a two-stage operation was necessary due to the rapid growth of his chondroblastoma. During the interim period, a cement spacer was inserted between the humerus and scapula while the custom implant was being manufactured. Patient 2 had a history of a chondroid lesion curettage 10 years prior the chondrosarcoma resection. Patient 3 had previously undergone several shoulder surgeries at another medical center. The initial procedure involved a Latarjet procedure, followed by multiple enchondroma curettages in subsequent surgeries. Patient 4 had an extensive medical and surgical history, encompassing issues such as pulmonary embolism, acute pericarditis, and deep-vein thrombosis. Additionally, he had undergone radiotherapy for his glenoid lesion. A standard radio-clinical post-operative follow-up facilitated the assessment of implant evolution. This evaluation involved a comparison of patients’ radiographs, ROM, and pain score as the Visual Analog Scale (VAS).

VAS and ROM measurements were recorded eight times during the follow-up period for patients who had undergone an initial reverse total shoulder arthroplasty. For patients who underwent totalization during follow-up, seven measurements were taken before totalization and an additional seven measurements were recorded afterward. ROM were measured from neutral position of the upper limb.

### 2.1 Statistical analysis

Statistical inferences were performed thanks to non-parametric Mann-Whitney Tests. All tests were two-tailed and the statistical significance threshold was set for *p*-values less than 0.05. Standard deviation is noted after a mean value following the symbol ±. All analyses were performed using SPSS software (V.27, SPSS, Inc., Chicago, IL, USA).

## 3 Results

Following a significant decline in joint amplitudes, increased pain, and progressive destruction of the humeral head visible on x-rays, both patients who had undergone glenoid hemiarthroplasty required revision surgery for humeral resurfacing. Patient 1 underwent resurfacing 7.79 months after glenoid reconstruction, while Patient 2 underwent resurfacing 33.60 months after glenoid reconstruction. Consequently, the clinical data for these patients has been merged with the total shoulder prosthesis group for ongoing analysis.

Two patients encountered medium- and long-term complications. Patient 4 experienced a prosthetic dislocation between the humeral stem and the reverse component at 13.15 months post-op, necessitating one-stage revision surgery to replace the reverse component on the Morse cone. This patient also presented a spontaneous fracture of the scapular spine attributed to the previous radiotherapy and the modifications in mechanical constrains of the shoulder. Patient 2 suffered implant dislocation one month after her shoulder was totalized, requiring reduction through open surgery.

We did not observe any infection or implant loosening.

### 3.1 Radiologic assessment

Radiologically, the hemiarthroplasties exhibited poor progression. Over the weeks, there was observable deformation and gradual loss of congruence in the proximal humerus and articular cartilage facing the implant. This concern regarding the anatomical destruction of the joint aligned completely with clinical observations with a decline in the patient's range of motion and an increase in pain.

**Figure 1:**
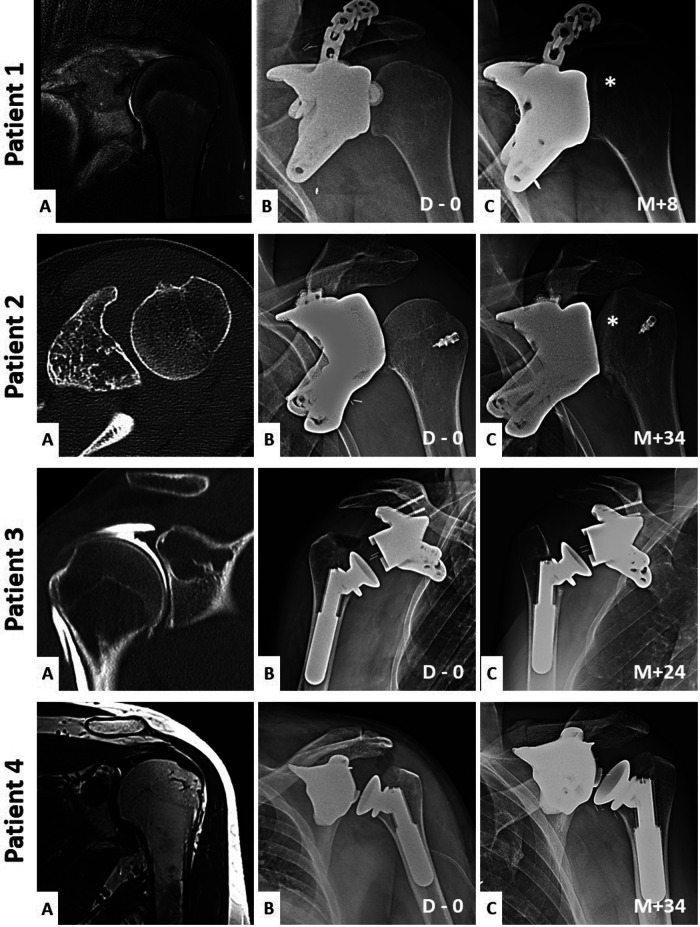
Preoperative and short and long-term imaging of the patient's shoulder. Patient **(1-A)** is a coronal T2 MRI sequence. Patient **(2-A)** is a coronal T1 MRI sequence. Patient **(3-A)** is a coronal injected arthro-CT acquisition. Patient **(4-A)** is an axial CT-scan acquisition. Each **(A)** images show the initial pathology. Each **(B)** images show the immediate postoperative x-rays of the shoulder's reconstructions (D = days). Each **(C)** images show the long-term postoperative x-rays of the reconstruction's evolutions (M = months). Asterisks show the humeral head deformations when a hemiarthroplasty is used.

Figure 1 shows the initial pathology and the evolution of short- and long-term implant imaging.

### 3.2 Function assessment

Upon revisiting the medical records, the most frequently ROM evaluated during post-operative follow-up included abduction, external rotation, internal rotation, and antepulsion. These assessments were conducted within the context of active movements. Descriptive statistics for these variables are presented in Table 1. Despite evident disparities in the descriptive data, statistically significant difference was identified between the two groups only for the internal rotation (*p*-value = 0.015). Abduction, external rotation and antepulsion showed no statistically significant difference (*p*-values = 0.806, 0.237 and 0.656 respectively). The considerable variability indicated by the standard deviation can be attributed to the anticipation of improvement in clinical examinations, thereby resulting in a lack of normality in the data.

**Table 1:** Descriptive statistics of the ROM assessment of the two groups from a neutral position: glenoid arthroplasty and total shoulder arthroplasty. Abduction, external rotation, internal rotation and antepulsion are expressed in degrees. Measures were recorded multiple times throughout the clinical follow-up.

Type of arthroplasty		Abduction (°)	External rotation (°)	Internal rotation (°)	Antepulsion (°)
Glenoid hemiarthroplasty	Mean ± std dev.	47 ± 27	7 ± 15	35 ± 35	60 ± 32
	[min–max]	[10–90]	[0–30]	[10–60]	[10–100]
Total shoulder arthroplasty	Mean ± std dev.	52 ± 18	21 ± 22	90 ± 11	67 ± 18
	[min–max]	[15–90]	[−10 to 60]	[60–110]	[40–110]
Total	Mean ± std dev.	51 ± 19	18 ± 21	83 ± 23	66 ± 22
	[min–max]	[10–90]	[−10 to 60]	[10–110]	[10–110]

### 3.3 Pain assessment

**Figure 2:**
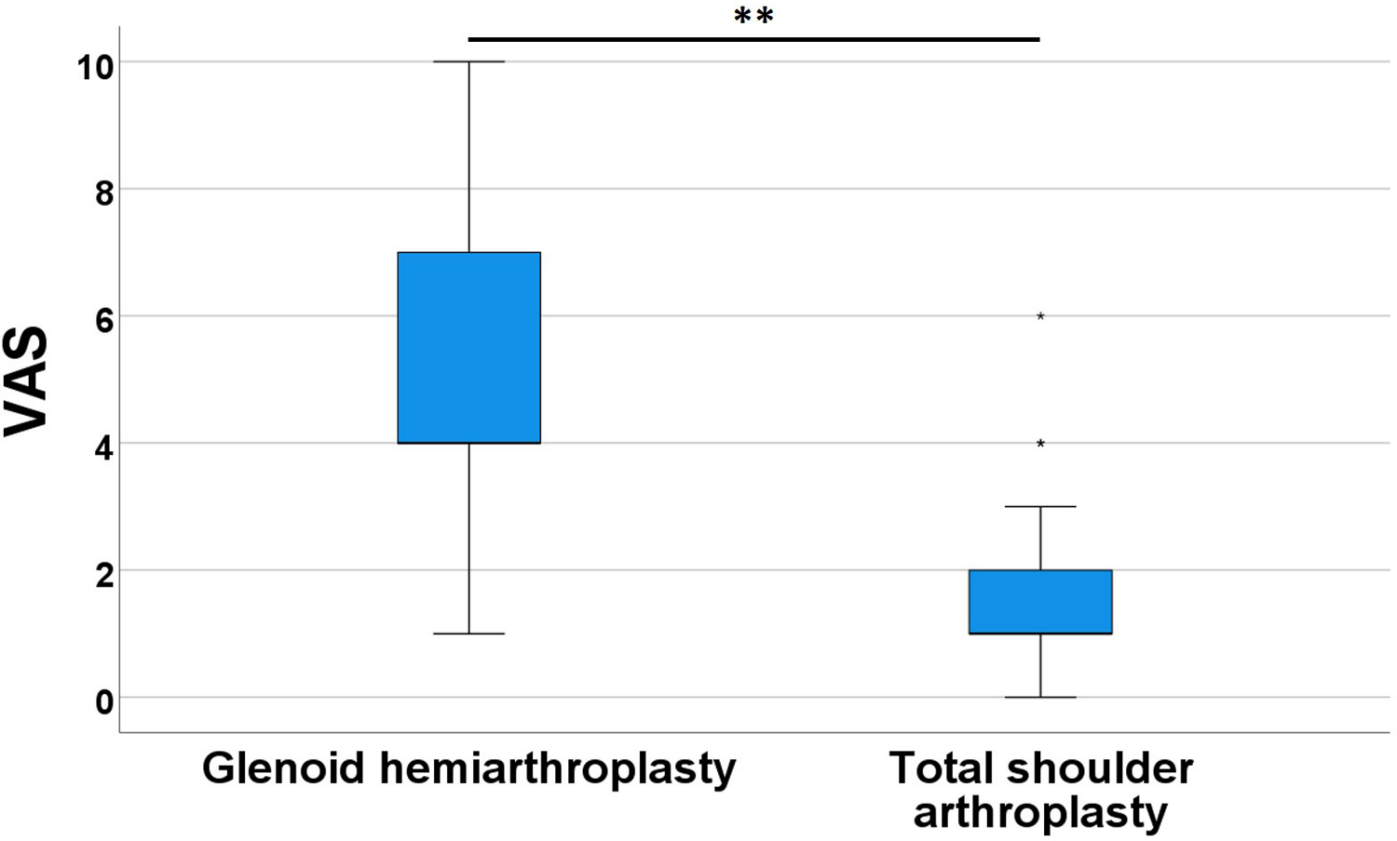
VAS boxplots comparing the pain scoring of glenoid hemiarthroplasty group to the total shoulder arthroplasty group. ** = *p*-value < 0.01. VAS were recorded multiple times throughout the clinical follow-up.

The Visual Analog Scale (VAS) scores, collected during postoperative consultations, provided a valuable insight into patients’ complaints. These scores potentially influenced the surgeon's decision towards revision surgery, transforming hemiarthroplasty into total arthroplasty. Upon comparing the VAS scores between the two groups, an average score of 5.1 ± 3.1 was observed for glenoid hemiarthroplasties, whereas total shoulder arthroplasties exhibited an average score of 1.5 ± 1.5. These means were found to be highly statistically significant (*p*-value = 0.002). Figure 2 visually depicts these central tendencies along with the variabilities in this variable.

### 3.4 Final outcomes

The clinical course of patients 1 and 2 deteriorated relatively rapidly with the deformation of the humeral head against the metal implant (Supplementary Figure S2a). This was expressed by a limitation of ROM and an increase in pain at rest and during movement. It was decided to resurface their proximal humerus, thus transforming a glenoid hemiarthroplasty into a custom-made anatomic total shoulder prosthesis for the subsequent follow-up (Supplementary Figure S3a). Patient 2 experienced one incident of dislocation following humeral resurfacing.

## 4 Discussion

This article conducts a comparison between two methods of shoulder reconstruction following oncological resection of the glenoid: glenoid hemiarthroplasty and total reverse arthroplasty. A radio-clinical evaluation of these two implant types revealed inferior outcomes associated with glenoid hemiarthroplasty (Figure 1).

x-rays clearly indicate the risks associated with relying solely on a glenoid implant. The decision of doing a reverse construct was based on two observations. Firstly, the suprascapular neurovascular bundle is inevitably either damaged or cut during this type of surgery. Either the bone cuts or the cutting jigs were always close by the anatomical landmarks of the bundle. This resulted invariably in the loss of the supra and infraspinatus function. A reverse construct makes therefore much more sense to compensate for the loss of function of a part of the rotator cuff.

**Figure 3:**
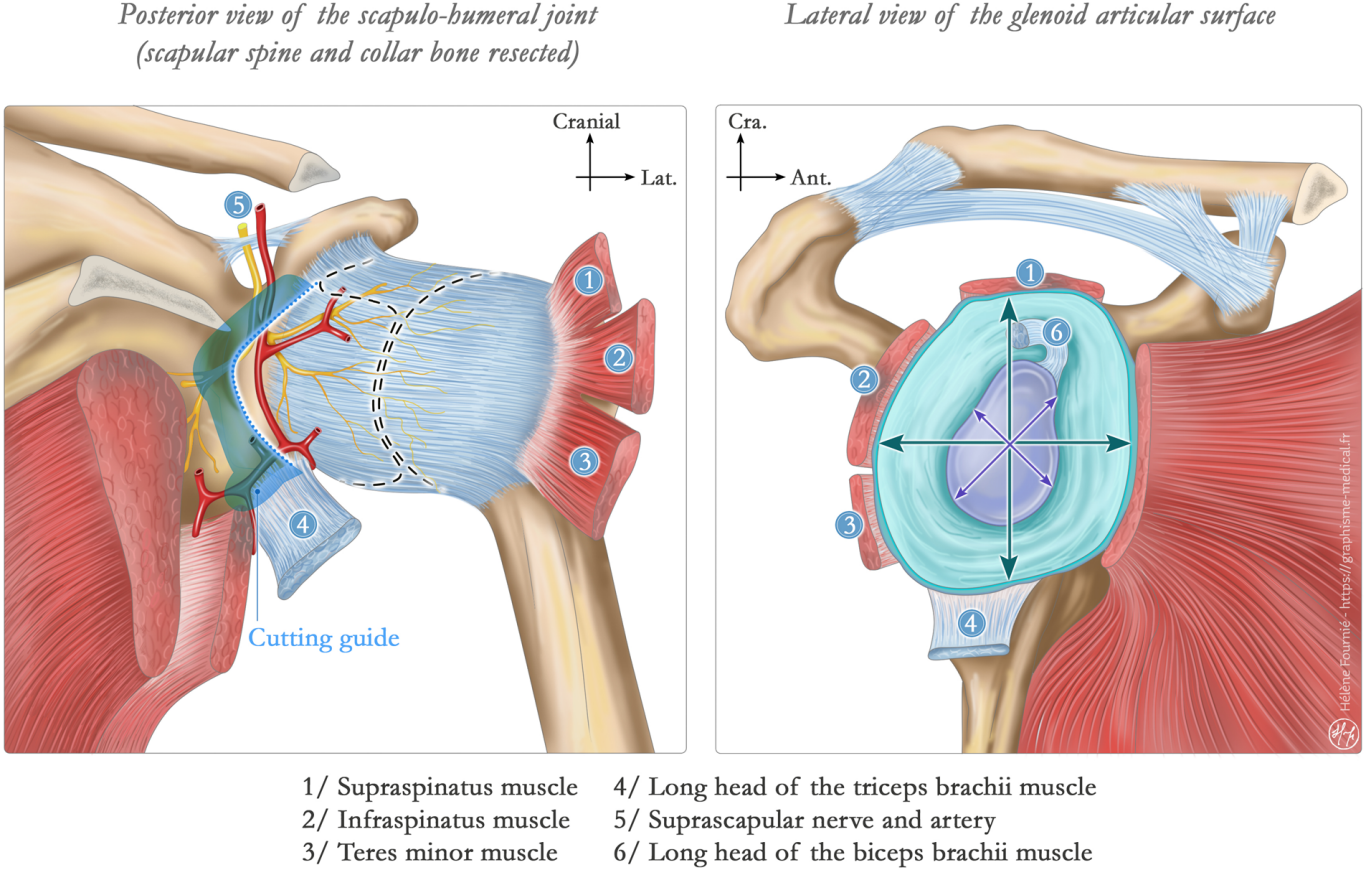
Illustration of a scapular glenoid surface. Left illustration shows the normal scapula-humeral joint in a posterior view. Articular surfaces are represented in dotted line within the articular capsule. The cutting guide in blue shows how the suprascapular pedicle might be compromised in this custom-made glenoid hemiarthroplasty. Right illustration shows the glenoid articular surface. The crosses show the discrepancy between the “bony articular surface” and the augmented articular surface with the labrum (turquoise structure) and other soft tissues. This last statement is the key to our findings of rapid humeral head destruction facing a glenoid hemiarthroplasty.

Secondly, a custom-made anatomical reproduction of the glenoid only reconstruct the bony aspects of articular surface but not the labrum. The surface of the bony glenoid is significantly smaller than the surface encompassing the bone and the labrum surface (Figure 3). Thus, the pressure of the glenoid on the humeral head is significantly increased resulting in a noticeable progression of subsidence and destruction on the humeral articular surface. Consequently, joint congruence is compromised, leading to a reduction in ROM and a significant increase in pain.

It is noteworthy that patient 2 exhibited humeral destruction much later than the revision surgery of patient 1 (M + 34 compared with M + 8). This delay is attributed to the fact that patient 2 underwent hemiarthroplasty just before the onset of the COVID-19 pandemic-related lockdown. Consequently, this patient remained without follow-up for over two years before undergoing surgery again due to a substantial decline in ROM and increased pain.

While the radiological and VAS results exhibit clear differences, the ROM analysis did not reveal a significant difference between the two types of reconstruction. This observation could be attributed to two primary reasons. Firstly, oncologic shoulder arthroplasties encounter a considerable functional challenge. In conventional arthroplasty, the inherent mobility of the shoulder is so extensive that achieving complete restoration of joint amplitudes through shoulder arthroplasties remains a difficult task. In our patients, the extent of the surgery is far more aggressive. Consequently, joint amplitudes tend to remain somewhat restricted regardless of the type of implant, contributing to reduced postoperative variability in ROM.

Patients initially undergoing hemiarthroplasty later underwent humeral resurfacing, eventually receiving a custom-made equivalent of an anatomical shoulder prosthesis. It is crucial to acknowledge a bias in this study—significant differences exist between anatomical and reverse prostheses. These distinctions, well-documented in the literature, encompass their primary indications, biomechanics, clinical outcomes, complication rates, and longevity (3, 5–7, 11, 16, 17). Despite sharing the overarching purpose of arthroplasty, these implants are not as directly comparable as desired. For instance, the reverse prostheses are known to be more stable and then more constrained. Conversely, the anatomical prostheses are less stable and less constrained, this might have been resulting in episodes of dislocation in patient 2 after her reintervention with the totalization of her arthroplasty. However, we chose to group these two types of total arthroplasty within the same study group solely to compare them with the glenoid hemiarthroplasty technique, aiming to emphasize the inferior functional outcomes of the latter in comparison to any form of total shoulder prosthesis.

Secondly, it is crucial to carefully consider some of our statistical results. Notably, when patients who had undergone hemiarthroplasty began to exhibit poor clinical outcomes due to humeral degradation, revision surgery was indicated. Once the prosthesis was converted to a total arthroplasty, their ROM and VAS assessments were included in the total shoulder replacement group for the rest of their follow-up. As a result, the hemiarthroplasty group contains fewer ROM and VAS measurements compared to the total arthroplasty group. Our statistical inferences must therefore be interpreted with caution, considering this bias. Nonetheless, the strong significance of VAS (as well as internal rotation measurements), along with the descriptive statistics and qualitative imaging variables, reassures us that a real difference exists between the two groups, supporting the preference for total arthroplasty over glenoid hemiarthroplasty.

A third limitation of our study is the small number of patients. With only 4 patients included, the sample size may appear limited. However, isolated glenoid tumors are exceedingly rare occurrences (1, 18–23). The recruitment of our four patients spanned over a period of 4 years. To provide context, the most recent extensive series of patients with scapular tumors, reported by Öztürk et al. in 2019, covered a 15-year retrospective analysis with 187 patients. In that series of patients, only one had an isolated scapular glenoid tumor (24). Given the rarity of this pathology, our case series is commendable, particularly as our findings contribute to advancements and modifications in our future surgical strategies and implant design for addressing this type of pathology.

To broaden our discussion to a more comprehensive management of patients, a wide range of perioperative variables (both pre- and post-operative) must be considered in every medical-surgical decision. These include factors such as functional demand, oncological status, comorbidities, and the integrity of the musculotendinous system, which is particularly critical in any type of shoulder reconstruction. This article focuses specifically on the anatomical, surgical, and conceptual aspects of managing glenoid tumors. In the case of our patients, it would have been beneficial to implement prehabilitation techniques, which have been shown to significantly impact post-operative outcomes in conventional surgeries (25). Another key factor we have considered is that, in most cases, younger patients warrant a more conservative approach to bone stock preservation. However, we aim to demonstrate that glenoid tumors may require deviation from this principle, as strict adherence could lead to major complications.

In conclusion, to further address the utility and potential of 3D printing for custom orthopedic implants, it is important to acknowledge that, like any emerging technology, it involves a significant learning curve. This requires the support of a responsive team of engineers familiar with the complexities of orthopedic reconstruction. Nevertheless, studies have shown that the use of 3D technologies—whether in the form of reconstructed models (26, 27) or mixed reality (28)—enhances the quality of surgical planning, ultimately leading to improved surgical outcomes. From both therapeutic and educational perspectives, this technology holds great promise for improving clinical outcomes for a wide range of patients.

Our observations on patients 1 and 2 prompted us to adjust our surgical approach, excluding a specific type of orthopedic reconstruction in the context of tumor pathologies affecting the scapular glenoid. To mitigate potential biases in our study and considering the rarity of this clinical entity, conducting a multicenter retrospective study involving institutions that have previously performed this form of hemiarthroplasty would be valuable. As far as we are aware, this study represents the first attempt to elucidate the clinical outcomes of custom 3D printed glenoid hemiarthroplasties in the context of reconstruction following glenoid tumor resection.

## 5 Conclusions

Glenoid custom-made 3D printed implants may be a valuable option in oncological reconstructions in carefully selected cases. The loading of the proximal humerus onto a custom-made bone reproduction of the glenoid leads to the relatively swift destruction of the humeral articular surface. Furthermore, the supraspinatus and the infraspinatus functions are systematically lost due to the inherent damage of the surgical approach and positioning of the cutting jigs. Therefore, in cases of isolated glenoid neoplasms where the preservation of the rest of the scapula may be beneficial, we recommend avoiding a hemiarthroplasty and opting for custom-made reverse shoulder prosthetic construct from the onset to address these challenges more effectively.

## Data Availability

The original contributions presented in the study are included in the article/Supplementary Material, further inquiries can be directed to the corresponding author.

## Supplementary material

Supplementary Figure S1a Scapular part of a custom-made reverse implant. Note that the second flange is positioned posterior for easier attachment on the main body of the implant.

Supplementary Figure S2a Per operative views of patient 2 showing the imprint of the glenoid component in the articular surface of the humerus. **(A)** Computed Tomography acquisition of the shoulder, in coronal plane showing the destruction of the articular surface. **(B)** Resected part of destructed proximal humerus. **(C)** Surgical approach with a direct view of the dislocated humeral head with the imprint of the custom glenoid component depicted in **(D)** showing the preoperative design of the implant with the small contact surface of the prosthetic glenoid reproducing only the bony aspects of the glenoid.

Supplementary Figure S3a Pre and postoperative x-rays of patient 1 and 2 shoulder. **(1-A)** and **(2-A)** show the last x-ray of those patients where the clinical evaluations were the worst. The destruction of the humeral head is noticeable. **(1-B)** and **(2-B)** show the resurfaced humeral head with an uncemented implant.
