# Supplementary figures and images for: Case Report: Custom made 3D implants for glenoid tumor reconstruction should be designed as reverse total shoulder arthroplasty
Source: Front Surg. 2024 Oct 16;11:1433692. doi: 10.3389/fsurg.2024.1433692 (PMC11521977; doi:10.3389/fsurg.2024.1433692)

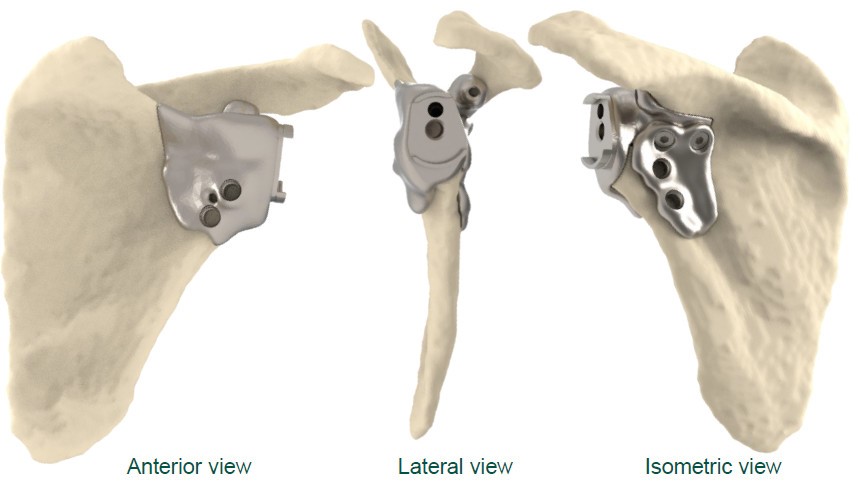

Supplement: Supplementary Figure S1a — Scapular part of a custom-made reverse implant. Note that the second flange is positioned posterior for easier attachment on the main body of the implant. [file Image1.jpg]

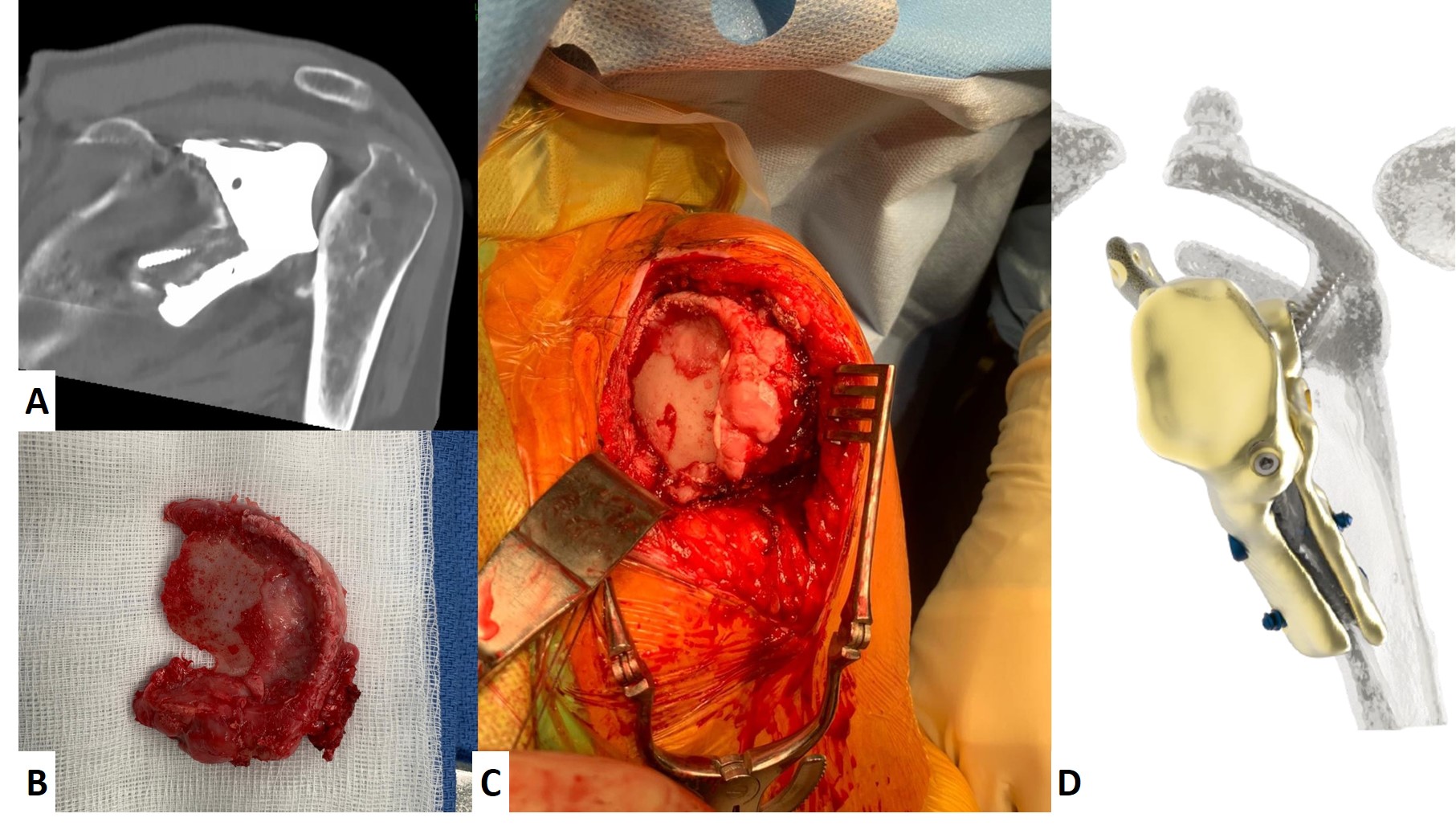

Supplement: Supplementary Figure S2a — Per operative views of patient 2 showing the imprint of the glenoid component in the articular surface of the humerus. (A) Computed Tomography acquisition of the shoulder, in coronal plane showing the destruction of the articular surface. (B) Resected part of destructed proximal humerus. (C) Surgical approach with a direct view of the dislocated humeral head with the imprint of the custom glenoid component depicted in (D) showing the preoperative design of the implant with the small contact surface of the prosthetic glenoid reproducing only the bony aspects of the glenoid. [file Image2.jpg]

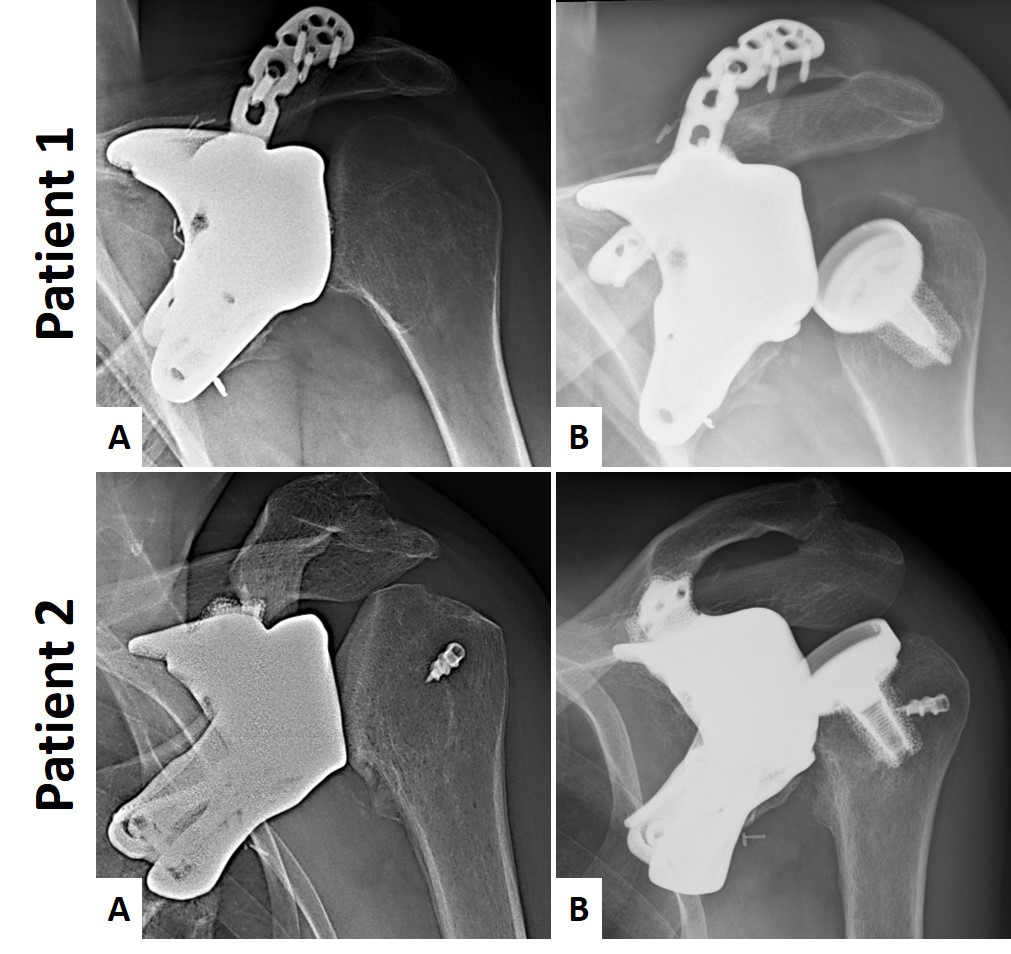

Supplement: Supplementary Figure S3a — Pre and postoperative x-rays of patient 1 and 2 shoulder. (1-A) and (2-A) show the last x-ray of those patients where the clinical evaluations were the worst. The destruction of the humeral head is noticeable. (1-B) and (2-B) show the resurfaced humeral head with an uncemented implant. [file Image3.jpg]
